# Hexokinase 2 is dispensable for T cell-dependent immunity

**DOI:** 10.1186/s40170-018-0184-5

**Published:** 2018-08-17

**Authors:** Manan M. Mehta, Samuel E. Weinberg, Elizabeth M. Steinert, Krishan Chhiba, Carlos Alberto Martinez, Peng Gao, Harris R. Perlman, Paul Bryce, Nissim Hay, Navdeep S. Chandel

**Affiliations:** 1 Department of Medicine, Northwestern University Feinberg School of Medicine, McGaw Pavilion, Rm. M-334, 240 East Huron Street, Chicago, IL 60611 USA; 2 Department of Biochemistry and Molecular Genetics, Northwestern University Feinberg School of Medicine, Chicago, IL 60611 USA; 3 Metabolomics Core Facility, Northwestern University Robert H. Lurie Comprehensive Cancer Center, Chicago, IL 60611 USA; 4 Department of Biochemistry and Molecular Genetics, University of Illinois at Chicago, Chicago, IL 60607 USA

**Keywords:** Hexokinase 2, T cells, Tregs, Leukemia, Colitis, LCMV

## Abstract

**Background:**

T cells and cancer cells utilize glycolysis for proliferation. The hexokinase (1–4) family of enzymes catalyze the first step of glycolysis. Hexokinase 2 (HK2) is one of the most highly upregulated metabolic enzymes in both cancer and activated T cells. HK2 is required for the development and/or growth of cancer in several cancer models, but the necessity of HK2 in T cells is not fully understood. The clinical applicability of HK2 inhibition in cancer may be significantly limited by any potential negative effects of HK2 inhibition on T cells. Therefore, we investigated the necessity of HK2 for T cell function. In order to identify additional therapeutic cancer targets, we performed RNA-seq to compare in vivo proliferating T cells to T cell leukemia.

**Methods:**

HK2 was genetically ablated in mouse T cells using a floxed *Hk2* allele crossed to CD4-Cre. CD4+ and CD8+ cells from mice were characterized metabolically and tested in vitro. T cell function in vivo was tested in a mouse model of colitis, Th2-mediated lung inflammation, and viral infection. Treg function was tested by crossing *Hk2*-floxed mice to FoxP3-Cre mice. Hematopoietic function was tested by deleting HK2 from bone marrow with Vav1-iCre. RNA-seq was used to compare T cells proliferating in response to virus with primary T-ALL leukemia induced with mutant Notch1 expression.

**Results:**

We unexpectedly report that HK2 is largely dispensable for in vitro T cell activation, proliferation, and differentiation. Loss of HK2 does not impair in vivo viral immunity and causes only a small impairment in the development of pathological inflammation. HK2 is not required for Treg function or hematopoiesis in vivo. One hundred sixty-seven metabolic genes were identified as being differentially expressed between T cells and leukemia.

**Conclusions:**

HK2 is a highly upregulated enzyme in cancer and in T cells. The requirement for HK2 in various cancer models has been described previously. Our finding that T cells are able to withstand the loss of HK2 indicates that HK2 may be a promising candidate for cancer therapy. Furthermore, we identify several other potential metabolic targets in T-ALL leukemia that could spare T cell function.

**Electronic supplementary material:**

The online version of this article (10.1186/s40170-018-0184-5) contains supplementary material, which is available to authorized users.

## Background

T lymphocytes in the immune system and neoplastic cells in cancer are both highly proliferative cell types, relying on highly active metabolic pathways [1, 2]. T cells typically divide and expand in response to a specific antigen, while cancer cells exhibit uncontrolled proliferation. Nonetheless, similar molecular mechanisms underlie the ability of these different cell types to proliferate. Indeed, one of the major side effects of chemotherapies which target proliferating cancer cells is immunosuppression. One common mechanism utilized by both cancer and activated T cells to support their proliferation is an upregulation of glycolysis to provide intermediates for macromolecule synthesis [3, 4].

Glucose metabolism is essential for both normal T cell-mediated immunity and pathological T cell-mediated inflammation [5–11]. Studies have similarly shown that glucose metabolism is required for tumor growth in a variety of cancer models [12]. Furthermore, in the tumor microenvironment, evidence indicates that T cells and cancer cells may compete for glucose and that cancer cells may upregulate glycolysis in part to deprive T cells of glucose as a means to evade and suppress the immune system [13, 14]. These studies have led to interest in using anti-glycolytic agents for cancer therapy. Clearly, glucose metabolism is essential for both cancer and T cells. However, it is not clear if glucose is utilized differently in cancer and T cells and thus if glucose metabolism is a viable target for cancer therapy. Given that T cells also rely heavily on glucose, it is possible that anti-glycolytic therapy could lead to immunosuppression, leaving patients susceptible to infections. Furthermore, such immunosuppression would work at cross purposes with emerging cancer therapies such as check point blockade which rely on T cells. Therefore, any potential glycolytic target for cancer therapy should ideally spare T cell function.

One possible target for anti-glycolytic cancer therapy is hexokinase (HK), first committed step of glycolysis. HK can be expressed as four different isoforms, with HK1 being a somewhat ubiquitous isoform and HK2 existing as a more selectively regulated isoform [15]. Cancer cells highly upregulate HK2 compared to their normal tissue of origin and it is required for tumorigenesis in a variety of mouse cancer models, including breast and lung cancer and T cell leukemia [16–19]. However, HK2 is also highly upregulated in activated T cells [20, 21]. Furthermore, pan-hexokinase inhibition with 2-deoxyglucose (2DG) causes impaired differentiation of the inflammatory Th17 CD4+ T cell lineage and a shift from effector to memory cells in CD8+ T cells [20, 22, 23]. Presently, it is unknown whether the loss of HK2 would impair T cell-mediated inflammation and immunity.

In this study, we report that HK2 is largely dispensable in T cells for viral immunity and inflammation in several mouse models. Collectively, our data indicate that targeting HK2 may be a promising avenue for further research in cancer therapy, as it may spare T cell function while inhibiting cancer growth.

## Methods

### Mouse models

HK2^fl/fl^ mice were previously generated [16]. HK2 WT, floxed (Fl), and null alleles were genotyped using the following primers: HK2-F (CCCCTTCGCTTGCCATTAC), HK2-R (TGTCTTGGCTCAGATGTGAC), and HK2-null (AACCACGACGCCCAATGATTTAG). CD4-Cre, Vav-iCre, FoxP3-YFP-Cre, CD45.1, and IL-10^−/−^ mice were obtained from the Jackson Labs. Except where noted otherwise, approximately 2–4-month old adult mice of both sexes were used for experiments. Animals were not randomized to experimental groups, but were age-matched, sex-matched, and littermates where possible. No animals were excluded from analysis. Investigators were blinded to mouse genotypes for histological scoring but not blinded otherwise. All animals were housed and bred in the Northwestern animal vivarium, and procedures were approved by the Institutional Animal Care and Use Committee (IACUC) at Northwestern University.

### T cell isolation and in vitro culture

Spleens from approximately 2–4-month old mice were mechanically disrupted through a 70 uM strainer in 2% FBS/PBS to yield splenocytes. CD4+ T cells and CD8+ T cells were isolated from splenocytes using a negative selection magnetic bead kit according to manufacturer’s protocol (StemCell). Resulting T cells were activated 1:1 with CD3/CD28-coated beads (Gibco) and 20 ng/mL IL-2 (PeproTech) for 24–72 h in RPMI 1640 media, 10% fetal bovine serum (FBS), 10 mM HEPES, 2 mM glutamax, and 50 uM beta-mercaptoethanol with antibiotic/antimycotic. Oxygen consumption and extracellular acidification rate were measured as previously descried using a Seahorse XF96 analyzer (Agilent) [24]. For proliferation experiments, cells were stained with CFSE prior to activation according to manufacturer’s protocol (Thermo Fisher). For low glucose experiments, dialysed fetal bovine serum was used and glucose was added as indicated. For experiments requiring conditions biased for certain lineages, activation was altered as follows. Cells were activated with 5:1 irradiated splenocytes (3000 rad) and 2.5 μg/mL soluble anti-CD3 (eBiosciences). Cytokines and blocking antibodies were added to promote Th1 (10 ng/mL IL-12, 10 μg/mL anti-IL-4), Th2 (300 ng/mL IL-4, 10 μg/mL anti-IL-12, 10 μg/mL anti-IFNγ), Th17 (2 ng/mL TGFb, 20 ng/mL IL-6), and Treg (2 ng/mL TGFb) differentiation. Th0 consisted of base media without any additional cytokines or blocking antibodies. IL-12 was obtained from PeproTech, anti-IL-4 and anti-IFNγ from BioLegend, IL-6 and anti-IL-12 from eBiosciences, and IL-4/TGFb from R&D. Cells split 1:2 after 3 days and supplemented with additional media and IL-2 for 2 days before analysis.

### Glucose assays

HK activity and 2-DG uptake were measured on activated CD4+ and CD8+ T cells by commercially available kits (BioVision and Promega, respectively) according to manufacturer’s instructions. HK lysates were flash frozen in liquid nitrogen prior to storage at − 80 °C.

### Flow cytometry and sorting

Single-cell suspensions were resuspended in 2% FBS/PBS for surface staining, and the following antibodies were used as indicated (clone numbers in parenthesis): anti-CD4 (GK1.5, RM4–5), CD8 (53–6.7), CD45.2 (104), CD25 (PC61.5), CD69 (H1-2F3), CD44 (IM7), CD62L (MEL14), F4/80 (BM8), CD11c (N418), B220 (RA3-6B2), CD11b (M1/70), Gr-1 (RB6-8C5), NK1.1 (PK136), TER-119 (TER-119), and CD19 (1D3). Viability was determined by exclusion of nucleic dye, either propidium iodide (Thermo Fisher), LIVE/DEAD Fixable Blue stain (Thermo Fisher), or Ghost Dye Red 780 (Tonbo). For intracellular cytokine staining, cells were pretreated with 50 ng/mL PMA (Sigma), 1μg/mL ionomycin (Sigma), and protein transport inhibitor cocktail (eBiosciences) for approximately 4 h (except in case of gp33 stimulation, which is performed as described below). For intracellular cytokines and transcriptions factors, cells were fixed with a Cell Fixation/Permeabilization Kit (BD) or FoxP3/Transcription Factor Staining Kit (eBiosciences) and stained with the following antibodies as indicated: anti-Tbet (O4–46), GATA3 (TWAJ), RORγt (B2D), FoxP3 (FJK-16 s), IFNγ (XMG1.2), IL-4(11B11), and IL-17a (17B7). Absolute counts were obtained by running samples with a known concentration of beads (Spherotech) or by counting cells on a Cellometer (Nexcelom). gp33 monomer (D(b)/LCMV.GP33.KAVYNFATM) was obtained from the NIH Tetramer Core Facility and tetramerized according to their provided protocol. All samples were run on LSR Fortessa or FACSymphony flow cytometers (BD) and data was analyzed using FlowJo software. Cells were sorted using the FASC Aria II (BD). Sorted cells were reanalyzed afterwards to ensure approximately 90% purity.

### Protein extraction and western blot

Cells pellets were stored at − 80 °C until resuspension and lysis. Protein content was quantified by BCA assay. Four to twenty percent or Any kD polyacrylamide gels (Bio-Rad) were used to separate lysate proteins. Protein was then transferred to nitrocellulose membranes using Trans-Blot Turbo (Bio-Rad). Membranes were blocked using 5% milk/TBS for 1 h, washed with TBST, and then incubated in 5% BSA/TBST with anti-HK2 (Santa Cruz, product #6521, diluted approximately 1:500) and anti-β-actin (Sigma, catalog no. T9026/A2066; diluted 1:1000) or anti-GAPDH (Santa Cruz, clone C65, diluted 1:1000) overnight. HK1 and HK3 blotting performed similarly with antibodies from Cell Signaling (clone C35C4) and Abcam (catalog no. ab126217), respectively. Membranes were washed with TBST and incubated in appropriate secondary antibodies—donkey anti-goat or goat anti-rabbit IRDye 800CW (LI-COR, diluted 1:5000-1:10000 in 5% milk/TBST) and donkey anti-mouse or anti-rabbit IRDye 680RD (LI-COR, diluted 1:10,000 in 5% milk/TBST)—for 1 h at room temperature. Membranes were imaged using the Odyssey Fc Analyzer (LI-COR).

### RNA isolation and real-time PCR

RNA was isolated using standard lab protocols. cDNA was generated using M-MLV Reverse Transcriptase (Invitrogen). Real-time PCR was performed on the CFX384 Real-Time System (Bio-Rad) using iQ SYBR Green Supermix (Bio-Rad) and 200 nM primers. Alternatively, low quantity RNA samples were analyzed using a one-step protocol (Tonbo CYBRFast 1-Step RT-qPCR Lo-Rox). Primers used as follows: HK2-F (GTGTGCTCCGAGTAAGGGTG), HK2-R (CAGGCATTCGGCAATGTGG), HK1-F (AACGGCCTCCGTCAAGATG), HK1-R (GCCGAGATCCAGTGCAATG), HK3-F (TGCTGCCCACATACGTGAG), HK3-R (GCCTGTCAGTGTTACCCACAA), Hmgcs2-F (GAAGAGAGCGATGCAGGAAAC), Hmgcs2-R (GTCCACATATTGGGCTGGAAA), Chdh-F (TTCGGCTGGATGGACATGAC), Chdh-R (CTGCTTACAAGTGTCTGGACC), Ldhb-F (CATTGCGTCCGTTGCAGATG), Ldhb-R (GGAGGAACAAGCTCCCGTG), Prodh-F (GCACCACGAGCAGTTGTTC), Prodh-R (CTTTGTTGTGCCGGATCAGAG), Fbp1-F (CACCGCGATCAAAGCCATCT), Fbp1-R (AGGTAGCGTAGGACGACTTCA), b-Actin-F (CTAAGGCCAACCGTGAAAAG), b-Actin-R RPL-19-F (GAAGGTCAAAGGGAATGTGTTCAA), and RPL-19-R (TTTCGTGCTTCCTTGGTCTTAGA). Data was analyzed using ΔCT method by comparing genes of interest to the geometric mean of indicated housekeeping genes.

### Colitis model and histology

IL10^−/−^ mice were allowed to spontaneously develop colitis, typically between 8 and 14 weeks of age. Mice were euthanized if they lost greater than 20% of their body weight or if they developed rectal prolapse. IL-10^+/+^ littermates were euthanized at approximately 13–15 weeks as controls. Colon, spleen, and mesenteric lymph nodes from euthanized mice were collected. Colons were fixed for 2 days in 10% neutral buffered formalin for H&E staining or immediately digested to isolate lamina propria cells as previously described [25]. Lymph nodes were mechanically disrupted in RPMI on scored plates to yield lymphocytes. Lymphocytes were analyzed by flow cytometry as described above. Fixed tissues were paraffin-embedded, sectioned, mounted, and stained with H&E by the Northwestern mouse histology core. Scoring was performed blinded according to established criteria [26].

### OVA immunization and airway inflammation

OVA immunization and challenge was performed as previously described [27]. Six to eight-week old mice were sensitized to OVA by treating with intraperitoneal 10 mg OVA (Grade VI, Sigma-Aldrich) in alum (3 mg) or equal volume PBS/alum on days 0 and 14. On days 21–23, mice were challenged daily with 1% OVA/PBS aerosolized by nebulizer. Mice were euthanized on day 24, trachea was cannulated, and lungs were flushed with PBS + 10% FCS + 1 mM EDTA. Resulting BAL fluid was counted for total nucleated cell count, cells were cytospun onto slides, and supernatants were used for ELISA cytokine quantification. Differential counts were performed after staining slides with DiffQuik. Lung tissue was homogenized for RNA isolation (Qiagen) and analyzed by TaqMan probes according to manufacturer’s protocol (Thermo Scientific, probe numbers mm00445259_m1, mm00439646_m1, mm00434204_m1, mm01168134_m1, and mm00439618_m1). Remaining lung tissue was fixed and H&E stained as described above.

### LCMV infection, CD8+ T cell restimulation, and plaque assay

Adult mice were infected with 200,000 pfu LCMV-Armstrong intraperitoneally. Peripheral blood was acquired by retro-orbital bleeds on indicated days. Red blood cells were lysed in RBC lysis buffer (eBiosciences) and blood was analyzed by flow cytometry as described above. Mice were euthanized after 60 days post infection (DPI) and splenocytes were isolated as described above. One million splenocytes per sample were stimulated with 30 ng/mL exogenous gp_33–41_ peptide (KAVYNFATM, GenScript) for approximately 5 h in the presence of GolgiPlug (BD), after which surface staining, tetramer staining, and intracellular cytokine staining were performed as described above. Alternatively, mice were euthanized at three and eight DPI, and liver and spleens were removed. Eight DPI spleens were sorted for CD8+ T cells which were used for RNA-seq and metabolomics as described below. Liver was homogenized and used to determine viral load by plaque assay as described in detail by others [28]. Briefly, serial dilutions of liver homogenate were added to a monolayer of Vero cells and overlayed with 0.5% agarose for 5 days, stained with neutral red for 1 day, and PFUs were counted.

### Bone marrow isolation and leukemic transformation

Peripheral blood counts were obtained by retro-orbital bleeds of mice and analysis on a HEMAVET (Drew). Bone marrow from adult mice was harvested by dissecting pelvis, femur, and tibia. Bones were centrifuged for 5 min at 5000 rcf in an 18G-punctured 500 uL tube nested in a 1.5 mL tube. Platinum-E cells were used to create Notch1-DE retrovirus by treating cells with MIGR1-Notch1-DE plasmid and using the jetPRIME manufacturer’s protocol (plasmid and cell line gifted by Panagiotis Ntziachristos). Bone marrow was used with a CD117+ positive selection magnetic bead isolation kit (StemCell) to yield CD117+ cells. CD117+ cells were grown in Opti-MEM (Thermo Fisher) supplemented with 10 ng/mL IL-3 (PeproTech), 10 ng/mL IL-7 (PeproTech), 50 ng/mL SCF (PeproTech), 50 ng/mL Flt3L (PeproTech), and 20 ng/mL IL-6 (eBiosciences). Cells were infected with Notch1-DE retrovirus by adding virus to cells, centrifugation at 2500 rpm at 25 °C for 90 min, incubation at 37 °C for 4 h, and removal of virus replacing with previous media. Infection was repeated on the next day, after which cells were allowed to rest for 2 days. Seventy-five thousand lineage (CD4, CD8a, B220, CD11b, Gr-1, NK1.1, Ter-119)-negative, GFP+ cells were sorted and mixed with 750,000 bone marrow cells from CD45.1 mice resuspended in PBS. Cells were adoptively transferred into lethally irradiated (1000 rad) CD45.1 mice by retro-orbital injection. Mice were monitored over 6 weeks by analyzing peripheral blood with flow cytometry for CD45.2 + GFP + CD4 + CD8+ leukemic cells to ensure development of leukemia. Splenocytes were sorted for leukemic cells and analyzed by RNA-seq (described below).

### RNA-seq data acquisition and analysis

RNA quality and quantity were measured using Agilent 4200 TapeStation using High Sensitivity RNA ScreenTape System (Agilent Technologies). SMART-Seq v4 Ultra Low Input RNA Kit (Takara Bio USA, Inc.) was used to generate full-length cDNA, and NextEra XT DNA sample preparation kit (Illumina Inc.) was used to prepare the final library. An Illumina NextSeq 500 (Illumina Inc.) was used to sequence library with single-end sequencing (1 × 75 cycles) using NextSeq 500 High Output reagent kit (Illumina Inc.). FASTQ reads were trimmed using Trimmomatic to remove end nucleotides with a PHRED score less than 30 and requiring a minimum length of 20 bp. Reads were then aligned to the mm10 genome using TopHat version 2.1.0 using the following options: --no-novel-juncs, --read-mismatches 2, --read-edit-dist 2, --max-multihits 20, and --library-type fr-unstranded. The generated bam files were then used to count the reads only at the exons of genes using htseq-count with the following parameters: -q -m intersection-nonempty -s no -t exon. The R package edgeR was used to determine significance of differentially expressed genes, only genes with > 2-fold change in expression and adjusted *p* value < 0.01 were considered significant and included in heatmap. Genes were filtered for involvement in metabolism based on the KEGG mmu01100 genes. Broad Institute GSEA software was used to perform a pre-ranked GSEA analysis using 3000 permutations and the Hallmark pathway database.

### Metabolomics

Cell pellets were flash frozen in liquid nitrogen and stored at − 80 °C until metabolite extraction. Samples were thawed and pellets were resuspended in ice cold 80% methanol, freeze-thawed three times, and centrifuged at 18,000 rcf for 10 min at 4 °C. Supernatants were transferred to new tubes, dried, resuspended, and volume-adjusted so 500,000 cells’ worth of metabolites were analyzed for targeted metabolomics. Samples were analyzed by High-Performance Liquid Chromatography and High-Resolution Mass Spectrometry and Tandem Mass Spectrometry (HPLC-MS/MS). Specifically, system consisted of a Thermo Q Exactive in line with an electrospray source and an Ultimate3000 (Thermo) series HPLC consisting of a binary pump, degasser, and auto-sampler outfitted with a Xbridge Amide column (Waters; dimensions of 4.6 mm × 100 mm and a 3.5-μm particle size). The mobile phase A contained 95% (vol/vol) water, 5% (vol/vol) acetonitrile, 20 mM ammonium hydroxide, 20 mM ammonium acetate, pH = 9.0; and B was 100% acetonitrile. The gradient was: 0–1 min, 15% A; 18.5 min, 76% A; 18.5–20.4 min, 24% A; 20.4–20.5 min, 15% A; and 20.5–28 min, 15% A with a flow rate of 400 μL/min. The capillary of the ESI source was set to 275 °C, with sheath gas at 45 arbitrary units, auxiliary gas at 5 arbitrary units, and the spray voltage at 4.0 kV. In positive/negative polarity switching mode, an m/z scan range from 70 to 850 was chosen and MS1 data was collected at a resolution of 70,000. The automatic gain control (AGC) target was set at 1 × 106 and the maximum injection time was 200 ms. The top five precursor ions were subsequently fragmented, in a data-dependent manner, using the higher energy collisional dissociation (HCD) cell set to 30% normalized collision energy in MS2 at a resolution power of 17,500. Data acquisition and peak extraction/integration were carried out by Xcalibur 4.0 software and TraceFinder 2.1 software, respectively (Thermo Fisher). Low quality peaks were omitted from analysis. Resulting ion counts were normalized to total ion counts per sample and analyzed using multiple *t* tests with a Benjamini-Kreiger false discovery rate of 10%.

### Statistical analyses

Data were analyzed statistically using Prism 7 (GraphPad), except RNA-seq data which was analyzed as described above. Statistical tests, error bar representations, *p* values, biological replicates, sample sizes, and independent experiments are specified in figure legends. Appropriate statistical tests were selected for each comparison based on type of data and assuming normality. Adjustments for multiple comparisons were performed when appropriate and are described in figure legends. Representative images of gels and histology are from at least three biological replicates. No outliers were removed from data analysis.

## Results

### HK2 is dispensable for T cells in vitro

**Fig. 1:**
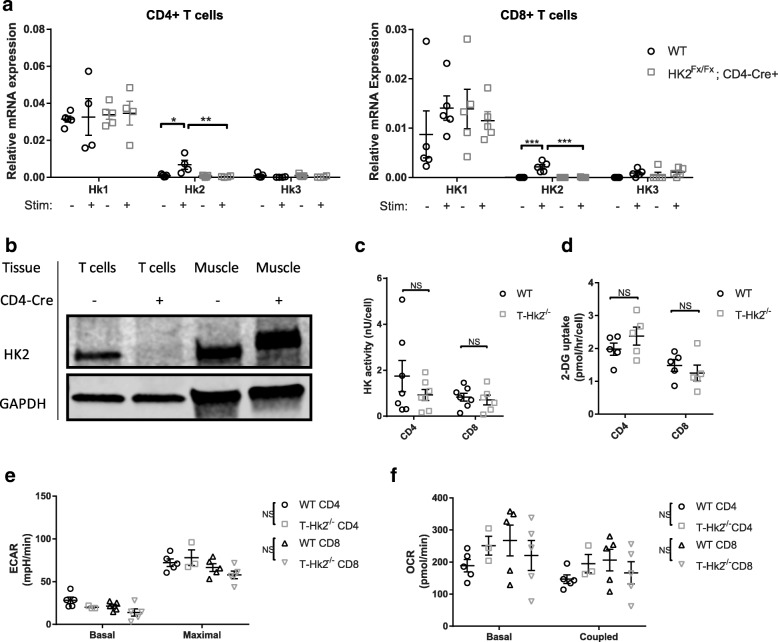
HK2 is dispensable for T cell glycolysis. Naïve CD4+ and CD8+ T cells were enriched from splenocytes isolated from adult WT and T-Hk2^−/−^ mice. T cells were activated in vitro with anti-CD3/28-coated beads for 24–72 h. **a** RNA expression of HK isoforms in CD4+ and CD8+ T cells after 24 h stimulation, compared to naïve unstimulated cells. Expression relative to β-actin and Rpl-19. Biological replicates, *n* = 4–5 (as indicated by individual data points) from at least two independent experiments **b** Western blot for protein expression of HK2 in muscle tissue or CD4+ T cells after 24 h stimulation. Representative of four independent experiments. **c**–**f** CD4+ or CD8+ T cells stimulated for 72 h and assayed for **c** total HK activity, **d** 2-DG uptake, **e** extracellular acidification rate (ECAR), and **f** oxygen consumption rate (OCR). **c**–**f** At least two independent experiments performed, biological replicates, *n* = 3–7 as indicated by individual data points. In all panels unless otherwise noted, error bars represent mean ± SEM, one-way or two-way ANOVA with Sidak multiple comparisons, *p < 0.05; **p < 0.01. NS = not significant

To assess the necessity of HK2 for basic T cell function in vitro, we generated mice that have a conditional deletion of the *Hk2* gene in T cells by crossing CD4-Cre mice with mice harboring a floxed *Hk2* allele (*Hk2*^fl/fl^). Resulting CD4-Cre, *Hk2*^fl/fl^ mice are herein referred to as T-*Hk2*^−/−^ mice. Spleens from the mice were removed and CD4+ and CD8+ cells were isolated and activated in vitro. As has been observed previously, CD4+ and CD8+ WT T cells highly upregulated *Hk2* transcript upon activation [20], but T cells from T-*Hk2*^−/−^ mice had no increase in HK2 mRNA or protein upon activation (Fig. 1a, b, Additional file 1: Figure S1A–B, Additional file 2: Figure S2A). Importantly, there was no compensatory increase in HK1 or HK3 mRNA or protein in CD4+ or CD8+ cells from T-*Hk2*^−/−^ mice (Fig. 1a, b, Additional file 1: Figure S1A–B). Surprisingly, total HK activity measured in activated CD4+ and CD8+ cells was not statistically different between WT and T-*Hk2*^−/−^ mice. (Fig. 1c). Glucose uptake capacity was measured by the amount of 2-DG taken up by cells. 2-DG uptake was unaffected by HK2 deficiency in CD4+ and CD8+ T cells (Fig. 1d). Basal and maximal extracellular acidification rate (ECAR) is a surrogate measure of lactate production and glycolytic activity. ECAR was similar between activated T cells from WT and T-*Hk2*^−/−^ mice (Fig. 1e). Oxygen consumption rate was also unchanged in activated CD4+ and CD8+ T cells (Fig. 1f). Overall, our data show that HK2 deficiency causes only small changes in T cell glucose usage.

**Fig. 2:**
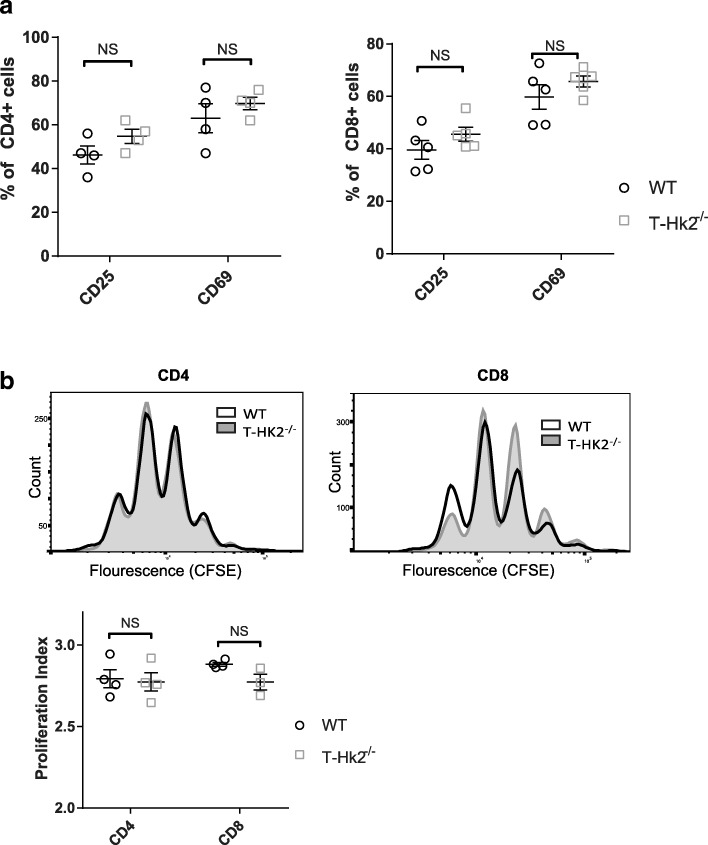
HK2 is not required for T cell activation and proliferation. Naïve CD4+ and CD8+ T cells were enriched from splenocytes isolated from adult WT and T-Hk2^−/−^ mice. T cells were activated in vitro with anti-CD3/28-coated beads for 24–72 h. **a** Percent of cells positive for indicated activation markers by flow cytometry gated on CD4+ (left) or CD8+ (right) cells after 24 h stimulation. Biological replicates, *n* = 4 from at least two independent experiments. **b** CFSE dilution in activated CD4+ and CD8+ T cells after 72 h of stimulation. Representative image of *n* = 4 biological replicates from at least two independent experiments. Quantified by proliferation index on right, *n* = 3–4 biological replicates as indicated by individual data points. In all panels unless otherwise noted, error bars represent mean ± SEM, one-way or two-way ANOVA with Sidak multiple comparisons, *p < 0.05; **p < 0.01. NS = not significant

In order to determine if HK2-deficient T cells had any functional deficits in vitro, we measured their ability to activate and proliferate after stimulation. Surprisingly, HK2-deficient CD4+ and CD8+ T cells had no decrease in expression of the activation markers CD25 or CD69 after 24 h (Fig. 2a, Additional file 3: Figure S3A). CD4+ T cells had no decrease in proliferation after 72 h as measured by CFSE staining, while CD8+ T cells consistently showed a small but insignificant deficit (Fig. 2b). Together, these data indicate that HK2 is not required for T cell viability, activation, or proliferation in vitro.

**Fig. 3:**
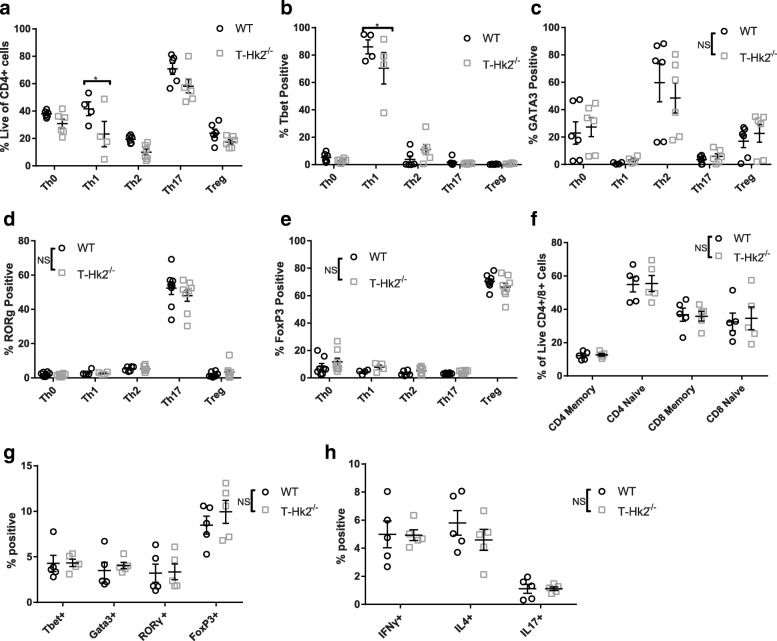
HK2 is dispensable for T cell differentiation. **a**–**e** Naïve CD4+ T cells were enriched from splenocytes isolated from adult WT and T-Hk2^−/−^ mice. Enriched naïve CD4+ T cells were stimulated with irradiated splenocytes under Th0, Th1, Th2, Th17, and Treg promoting conditions for 5 days. Percent of CD4+ T cells positive for viability or viable and positive for indicated transcription factor shown. Biological replicates, *n* = 4–8 (as indicated by individual data points) from at least two independent experiments. **f**–**h** Splenocytes were isolated from 8 to 12 week old WT and T-Hk2^−/−^ mice to analyze steady state levels of various populations. **f** Percent of viable CD4+ or CD8+ cells that are CD62L^hi^CD44^lo^ (naive) or CD44^hi^ (memory). Biological replicates, *n* = 5 mice. **g**–**h** Percent of viable CD4+ T cells positive for the indicated transcription factor (**g**) or cytokine (**h**). Biological replicates, *n* = 5 mice. In all panels unless otherwise noted, error bars represent mean ± SEM, two-way ANOVA with Sidak multiple comparisons, *p < 0.05; **p < 0.01. NS = not significant

Another important function of T cells is their ability to differentiate into specialized subsets. We tested the ability of CD4+ cells to differentiate in vitro by skewing them towards Th1, Th2, Th17, and Treg lineages. No major impairment was observed between WT- and HK2-deficient T cells in any of these lineages as determined by viability and expression of lineage defining transcription factors (Fig. 3a–e, Addtional file 3: Figure S3C). We also compared the steady state levels of different cell types in the spleens of WT and T-*Hk2*^−/−^ mice. We observed no differences in percent of CD4+, CD8+, naïve (CD62L^hi^CD44^lo^), memory (CD44^hi^), Th1, Th2, Th17, or Treg cells in T-*Hk2*^−/−^ mice (Fig. 3f–h, Additional file 4: Figure S4F, Additional file 3: Figure S3B). Furthermore, there were no observed differences in other immune cell numbers (Additional file 4: Figure S4G). Together, these data demonstrate that T-*Hk2*^−/−^ mice do not have an impairment in T cell differentiation in vitro or at steady state in vivo*.*

The unexpected dispensability for HK2 in vitro lead us to hypothesize that perhaps HK2 is upregulated in T cells to maintain glycolysis during conditions of low glucose availability. To this end, we activated CD4+ T cells in vitro under various glucose concentrations, from 5.5 mM (physiological blood glucose) to 0 mM. Previous work has shown that activation and viability at 24 h should not be affected by low concentrations of glucose, but the complete absence of glucose will cause T cell death that can be rescued by pyruvate [24, 29]. Consistent with previous findings, we observed a sharp decline in viability and activation in WT T cells between 0.1 mM glucose and 0 mM glucose that was restored with 1 mM pyruvate (Additional file 4: Figure S4A–B). Remarkably, HK2-deficient T cells exhibited an identical response to glucose concentrations as WT T cells (Additional file 4: Figure S4A–B). Similarly, WT- and HK2-deficient T cells stimulated for 3 days under varying concentrations of glucose showed comparable viability, proliferation as measured by CFSE proliferation index, and proliferation as measured by cell number (Additional file 4: Figure S4C–E). These data demonstrate that *Hk2* is dispensable in vitro for T cell viability, activation, and proliferation even under limiting glucose levels.

### HK2 deficiency mildly reduces T cell-mediated inflammation in vivo

**Fig. 4:**
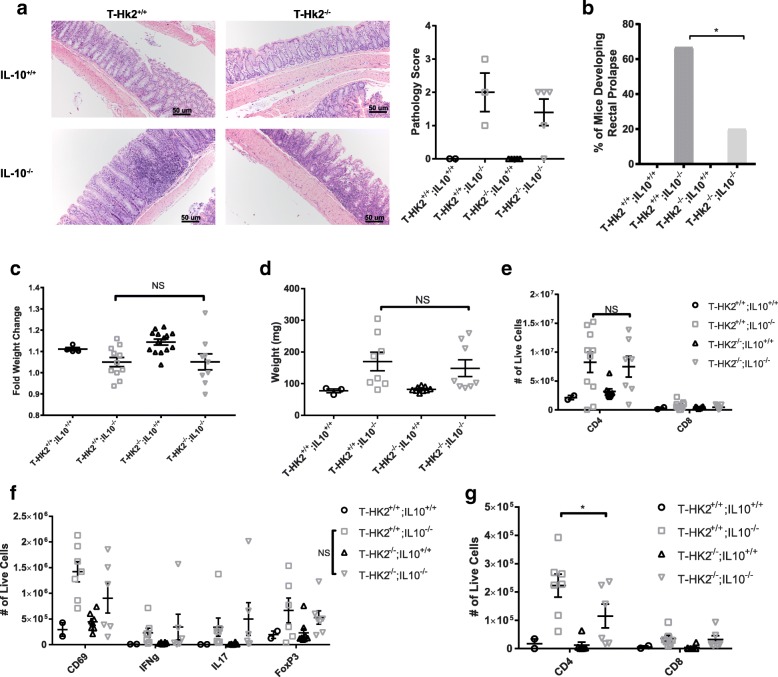
HK2 deficiency reduces T cell-mediated colon inflammation in vivo. T-Hk2^−/−^ mice were crossed to IL10^−/−^ mice to induce colitis. Mice were euthanized at approximately 13–15 weeks of age or earlier if required due to weight loss or rectal prolapse. **a** H&E staining and scoring of large intestine section in indicated mice. Scale bars show 50 um, representative images shown. Biological replicates, *n* = 2–5 mice as indicated by individual data points. **b** Percentage of mice which spontaneously develop rectal prolapse by 15 weeks of age. Chi-squared test. *n* = 4, 12, 10, 10 mice (left to right). **c** Fold change in weight between week 9 and euthanasia. Biological replicates, *n* = 4–15 mice, as indicated by individual data points. **d** Spleen weight. Biological replicates, *n* = 3–11 mice as indicated by individual data points. **e**-**g** Total number of indicated cell type isolated from mesenteric lymph nodes (**e**, **f**; *n* = 2–10 mice, as indicated by individual data points) and colon lamina propria (**g**; *n* = 2–7 mice, as indicated by individual data points). In all panels unless otherwise noted, error bars represent mean ± SEM, at least three independent experiments performed, one-way or two-way ANOVA with Sidak multiple comparisons correction, *p < 0.05; **p < 0.01. NS = not significant

It is possible that though we did not see any differences in vitro from loss of HK2, there could be differences in vivo in T-*Hk2*^−/−^ mice under inflammatory conditions. We tested whether HK2 deficiency in T cells would impair T cell-mediated inflammation by using a mouse model of colitis driven by IL-10 deficiency. IL10^−/−^ mice spontaneously develop colitis in a T cell-dependent manner thought to be driven by Th1 and Th17 cytokine-producing cells [30–33]. We generated *Hk2*^fl/fl^, CD4-Cre^+^, and IL-10^−/−^ mice that were globally deficient in IL-10 and had a T cell-specific HK2 deficiency (herein referred to as IL-10^−/−^; T-*Hk2*^−/−^). IL-10^−/−^ mice and IL-10^−/−^;T-*Hk2*^−/−^ mice both developed mild colitis marked by lymphocytic infiltrate in the colon, a failure to gain weight, splenomegaly, and rectal prolapse (Fig. 4a–d). IL-10-sufficient littermate control mice showed no signs of disease. Interestingly, IL-10^−/−^,T-*Hk2*^−/−^ mice developed spontaneous rectal prolapse at a significantly lower rate than IL-10^−/−^ mice, though weight change and splenomegaly were similar between the two (Fig. 4b–d). To further investigate differences in the inflammation between IL-10^−/−^ mice and IL-10^−/−^;T-*Hk2*^−/−^ mice, we analyzed tissues by flow cytometry which showed increase cellularity in the mesenteric lymph nodes and lamina propria of both IL10^−/−^ mice and IL-10^−/−^;T-*Hk2*^−/−^ when compared to IL10^+/+^ mice (Fig. 4e, f). There were no observed differences in CD4+, CD8+, CD69+, IFNγ +, IL17+, or FoxP3+ cell numbers in the mesenteric lymph nodes of IL10^−/−^ mice and IL-10^−/−^;T-*Hk2*^−/−^ mice (Fig. 4e, f). However, there was a mild decrease in CD4+ T cells in the lamina propria of IL-10^−/−^;T-*Hk2*^−/−^ mice (Fig. 4g). Overall, these data indicate that IL-10^−/−^;T-*Hk2*^−/−^ develop colitis, but with a lower severity than IL10^−/−^ mice.

**Fig. 5:**
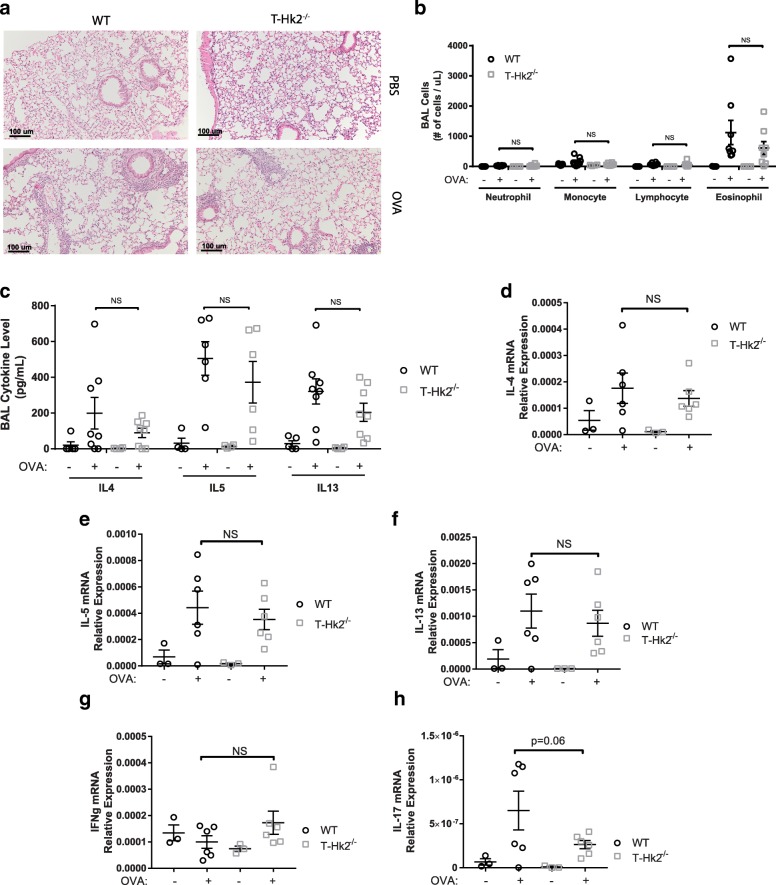
HK2 deficiency impairs Th2-mediated lung inflammation in vivo. Six to eight-week old WT and T-Hk2^−/−^ mice were pre-sensitized to OVA twice, 14 days apart with intraperitoneal injection of OVA/alum or PBS/alum. Twenty-one days after initial pre-sensitization, mice were challenged with aerosolized OVA daily for 3 days. Mice were euthanized for analysis on the fourth day. **a** H&E staining of lung tissue. Scale bars show 100 um, representative images shown from *n* = 4 mice. **b** Number of indicated cells present in BAL fluid (**c**) ELISA for indicated cytokines in BAL fluid. **b**, **c** Multiple *t* tests with Holm-Sidak correction for multiple comparisons. **d**–**h** RNA expression of indicated cytokine relative to β-actin. In all panels unless otherwise noted, error bars represent mean ± SEM, biological replicates, *n* = 5, 8, 4, 8 mice (left to right) from three independent experiments, unless otherwise noted, one-way ANOVA with Sidak multiple comparisons correction, *p < 0.05; **p < 0.01. NS = not significant

We also tested the necessity of HK2 in a mouse model of Th2 lung inflammation by sensitizing WT and T-*Hk2*^−/−^ mice with ovalbumin/aluminum hydroxide (OVA/alum) and provoking airway inflammation subsequently with aerosolized OVA. Inflammation in the airway in response to OVA is known to be a Th2-driven process with a Th17 component [34]. Non-sensitized WT and T-*Hk2*^−/−^ mice lacked any pulmonary inflammation after challenge with OVA while both WT and T-*Hk2*^−/−^ sensitized mice developed significant inflammation (Fig. 5a). Bronchoalveolar lavage after OVA challenge showed both WT and T-*Hk2*^−/−^ mice had an increase in BAL cellularity when compared to mice that were not previously sensitized (Fig. 5b). Absolute levels of T cells and eosinophils were increased in the BAL of both WT and *Hk2*^−/−^ mice, but T-*Hk2*^−/−^ mice had a small decrease in eosinophils when compared to WT mice (Fig. 5b). Th2 cytokines in the BAL were increased in both WT and T-*Hk2*^−/−^ mice (Fig. 5c). While T-*Hk2*^−/−^ mice had cytokine levels that trended downward compared to WT mice, the decrease was not statistically significant. Whole lung RNA cytokine levels showed similar results with a significant increase in Th2 and Th17 cytokines in both WT and T-*Hk2*^−/−^ mice when compared to unsensitized mice; IFNγ, a Th1 cytokine, was not increased (Fig. 5d–h). Furthermore, similar to BAL levels, RNA levels showed a descending trend in cytokine levels when comparing T-*Hk2*^−/−^ mice to WT mice. However, again, the decrease in cytokine levels of T-*Hk2*^−/−^ mice was not statistically significant, though the decrease in IL-17 approached significance (*p* = 0.06). Taken together, these results indicate that *Hk2* is not strictly required for development of Th2 inflammation, but its presence may modulate the extent of inflammation.

### HK2 is not required for T cell-mediated viral immunity

**Fig. 6:**
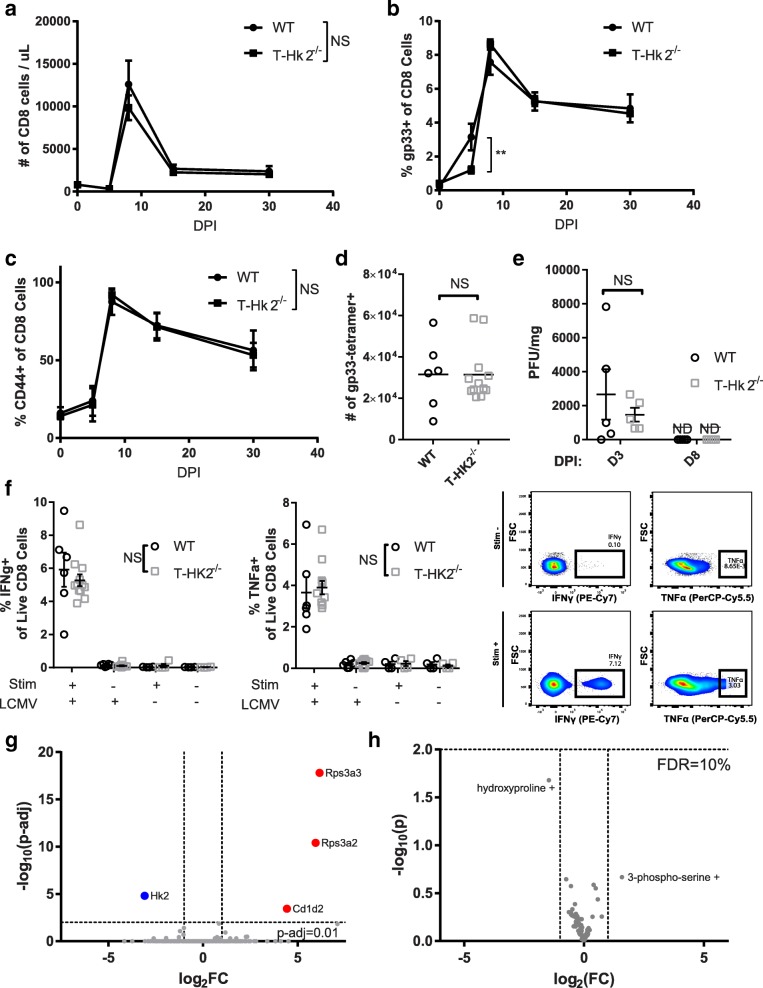
HK2 is largely dispensable for immunity to LCMV. Eight to twelve-week old WT and T-Hk2^−/−^ mice were injected intraperitoneally with 2 × 10^5^ pfu LCMV. **a**–**c** T cell expansion was monitored over time by flow cytometry of peripheral blood. **a** Number of CD8+ cells. **b** Percentage of CD8+ cells positive for gp-33 tetramer (D(b)/LCMV.GP33.KAVYNFATM) or (**c**) CD44. Biological replicates, *n* = 6 WT, 14 T-Hk2^−/−^ mice from three independent experiments. **d** Number of gp33-tetramer+ CD8+ T cells in spleen of WT and T-Hk2^−/−^ mice at 60 DPI. Biological replicates, *n* = 6 WT, 12 T-Hk2^−/−^ mice from two independent experiments, analyzed by unpaired Student’s *t* test. **e** Viral load as measured by plaque assay from right lobe of liver from 3 and 8 DPI. Biological replicates, *n* = 5 mice from two independent experiments. **f** Splenocytes from uninfected mice and mice 60 DPI were stimulated in vitro for 5 h with 30 ng/mL gp_33–41_ peptide or vehicle control and stained for IFNγ and TNFα secretion. Example gating shown on right. Biological replicates, *n* = 4 mice from two independent experiments. **g**, **h** RNA and metabolites were isolated from sorted splenic CD8+ T cells from WT and T-Hk2^−/−^ mice 8 DPI. **g** Volcano plot of gene expression differences. Differentially regulated genes include those with greater than 2 fold change in expression and multiplicity adjusted *p* value < 0.01. **h** Volcano plot of metabolite concentration differences. Vertical dashed lines indicate 2 fold change in metabolite concentration. Horizontal dashed line indicates no discoveries made (False Discovery Rate of 0.1, two-stage Benjamini-Krieger method, *n* = 5 mice). In all panels unless otherwise noted, error bars represent mean ± SEM, two-way ANOVA with Sidak multiple comparisons correction (**a**–**c** includes repeated measures), *p < 0.05; **p < 0.01. NS = not significant. n.d. = not detected. DPI = days post injection

To test the role of HK2 in viral immunity, we infected T-*Hk2*^−/−^ mice intraperitoneally with lymphocytic choriomeningitis virus (LCMV). Rapid clearance of the virus after acute infection requires function of CD8+ T cells and maintenance of a LCMV-specific memory population requires CD4+ and CD8+ T cell function [35, 36]. As we observed a small difference in proliferation of CD8+ T cells in vitro, we hypothesized there could be a defect in the anti-viral proliferative response, but T-*Hk2*^−/−^ mice show an antigen-specific CD8+ T cell expansion following infection with LCMV identical to that of WT mice; CD8+ T cells expand rapidly and then slowly contract (Fig. 6a). However, the percentage of CD8+ cells specific for the gp33 epitope of LCMV in the blood lags in T-*Hk2*^−/−^ mice compared to WT mice at 5 DPI, though it catches up by 8 DPI and remains at comparable levels for at least 60 days (Fig. 6b, Additional file 3: Figure S3D). Notably, the T-*Hk2*^−/−^ mice memory population expands and contracts just as the respective population in WT mice (Fig. 6c). Numbers of antigen-specific memory cells in the spleen are also similar between WT and T-*Hk2*^−/−^ mice at 60 DPI (Fig. 6d) and T-*Hk2*^−/−^ mice were able to clear infection by 8 DPI just as WT mice and had a similar viral load at 3 DPI (Fig. 6e). Furthermore, antigen-specific memory cells at 60 DPI are also functional, as they retain their ability to secrete IFNγ and TNFα following restimulation with exogenous gp33 peptide (Fig. 6f).

In order to determine if there were any in vivo differences in metabolism between WT and T-*Hk2*^−/−^ mice, we analyzed metabolite and transcript profiles of CD8+ T cells from the spleens of mice at 8 DPI. We found no significant difference in metabolite levels and only four differentially expressed genes between WT and T-*Hk2*^−/−^ mice (Fig. 6g, h). Predictably, of the few genes that were differentially expressed, *Hk2* stood out as the most downregulated gene in CD8+ T cells from T-*Hk2*^−/−^ mice. Importantly, HK1 was unchanged in both WT- and HK2-deficient T cells. These data indicate that HK2 is dispensable for CD8+ T cell-mediated viral immunity.

### HK2 is not required for Treg function in vivo

**Fig. 7:**
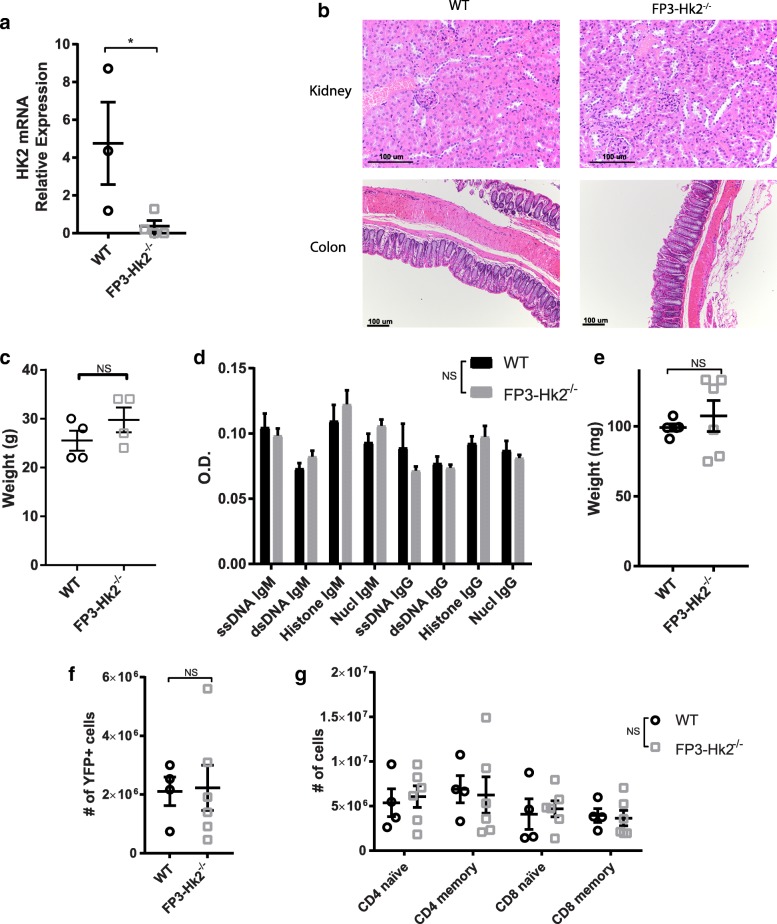
HK2 is not required for Treg function in vivo. Mice with YFP+ HK2-deficient Treg cells were generated (FP3-Hk2^−/−^ mice). **a** CD4+ cells from spleens of adult WT and FP3-Hk2^−/−^ mice were stimulated under Treg promoting conditions and RNA was isolated from sorted YFP+ cells. Hk2 expression relative to β-actin (*n* = 3 WT, 4 FP3-Hk2^−/−^). **b**–**g** WT and FP3-Hk2^−/−^ mice were aged to 6 months. **b** Representative H&E stained sections of indicated tissues, *n* = 4 mice. Scale bars show 100 um. **c** Mouse weight; biological replicates, *n* = 4 mice. **d** Titer of indicated auto-antibodies in serum; biological replicates, *n* = 4 WT, 7 FP3-Hk2^−/−^ mice. **e** Spleen weight; biological replicates, *n* = 5 WT, 6 FP3-Hk2^−/−^ mice. **f** Number of splenic Treg cells and **g** percent of viable CD4+ or CD8+ T cells that are CD62L^hi^CD44^lo^ (naive) or CD44^hi^ (memory); biological replicates, *n* = 4 WT, 6 FP3-Hk2^−/−^ mice. In all panels unless otherwise noted, error bars represent mean ± SEM, experiments performed twice independently, single-tailed Student’s *t* test or two-way ANOVA with Sidak multiple comparisons correction, *p < 0.05; **p < 0.01. NS = not significant

Although HK2 is not necessary for inflammatory CD4+ and CD8+ lineages, we next determined if HK2 was required for the function of the regulatory T cell lineage (Treg cells). We generated mice with Treg-specific deletion of the *Hk2* gene by crossing *Hk2*^fl/fl^ mice with FoxP3-YFP-Cre mice. Tregs isolated from these mice (FP3-*Hk2*^−/−^) did not show appreciable levels of *Hk2* mRNA transcript (Fig. 7a, Additional file 3: Figure S3E). FP3-*Hk2*^−/−^ mice did not develop any indications of autoimmune disease as would be expected of mice with deficiencies in Treg function [37, 38], as determined by histology, serum ANA titers, weight, and presence of splenomegaly (Fig. 7b-e). Furthermore, they had normal numbers of Tregs in the spleen, as well as normal numbers of naïve and memory CD4 and CD8 T cells (Fig. 7f, g). These data indicate that HK2 is dispensable for Treg function in vivo.

### HK2 is not required for normal hematopoiesis

**Fig. 8:**
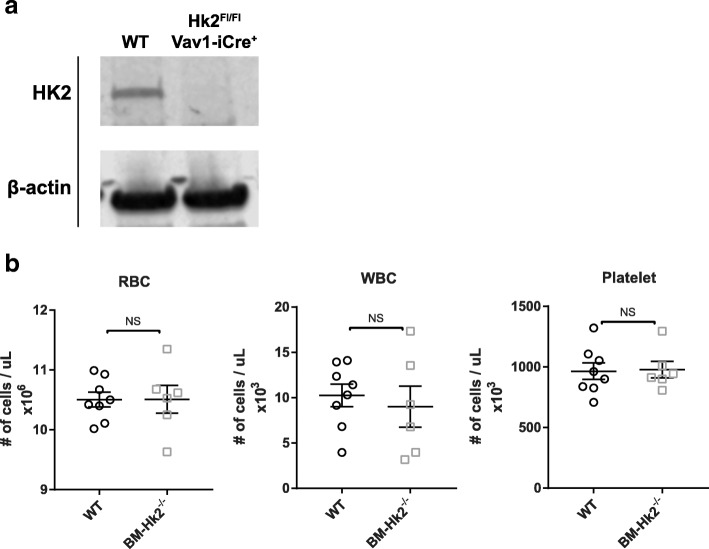
HK2 is not required for normal hematpoiesis. Mice with HK2-deficient bone marrow were generated (Hk2^Fl/Fl^;Vav1-iCre^+^, referred to as BM-Hk2^−/−^) **a** Western blot for protein expression of HK2 and β-actin in bone marrow. Representative of four independent experiments **b** Peripheral blood counts of indicated hematopoietic lineages in adult WT and Hk2^Fl/Fl^; Vav1-iCre^+^ mice. *n* = 8 WT, 6 Hk2^Fl/Fl^; Vav1-iCre^+^ mice. In all panels, biological replicates shown, at least two independent experiments performed, error bars represent mean ± SEM, unpaired *t* test, *p < 0.05; **p < 0.01. NS = not significant

To determine if there is any defect in the ability of other hematopoietic lineages to differentiate without HK2, we abolished HK2 function in hematopoietic stem cells by crossing *Hk2*-floxed mice with Vav1-iCre, which expresses Cre in hematopoietic stem cells during development [39]. Resulting BM-*Hk2*^*−/−*^ mice did not have any significant HK2 expression as determined by western blot (Fig. 8a, Additional file 2: Figure S2B). Furthermore, HK2 deficiency did not impair hematopoiesis as assessed by platelet, white, and red blood cell counts (Fig. 8b). Collectively, these data indicate HK2 is not required for normal hematopoiesis.

### Transcriptome differences between T cells and T cell leukemia in vivo

**Fig. 9:**
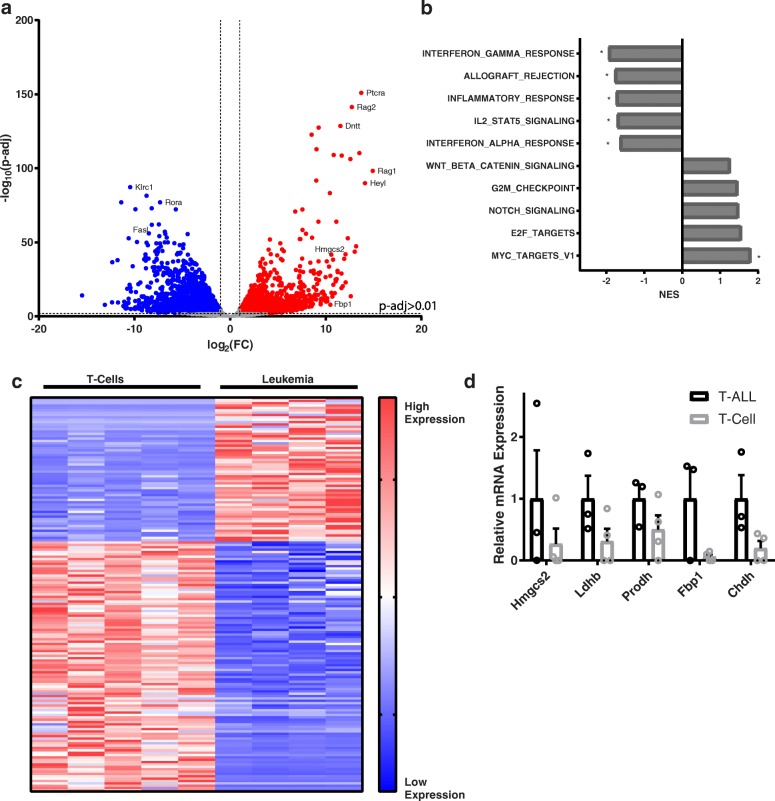
Transcriptomic and metabolomic analysis reveals essential differences in metabolism of leukemia and lymphocytes. Sorted splenic CD8+ T cells from WT mice 8 days after LCMV infection, compared to sorted T-ALL cells from WT mice 6 weeks after adoptive transfer. RNA isolated from sorted cells and analyzed by RNA-seq **a** Volcano plot of gene expression differences. Differentially regulated genes include those with greater than 2 fold change in expression and multiplicity adjusted *p* value < 0.01. Blue dots are overexpressed in CD8+ T cells, red in T-ALL. **b** Hallmark pathways enriched in leukemia (positive values) and T cells (negative values) and their normalized enrichment score. Statistical significance shown by * (FWER *p* < 0.05). **c** Expression of differentially regulated metabolic genes from the KEGG database. **d** Expression of select metabolic genes by RT-PCR relative to β-actin and Rpl-19. Biological replicates, *n* = 3–4 mice, as indicated by individual data points

While cancer cells and T cells both upregulated HK2 expression, our data indicate it is only required in the former. For example, HK2 deficiency has been shown to decrease tumor burden in a mouse model of T cell acute lymphoblastic leukemia (T-ALL) [19]. Finding therapeutics that target neoplastic T cells while sparing normal T cells represents an interesting challenge in cancer therapy, as the two share a common origin and common proliferative goals. In order to identify other potential metabolic targets for T-ALL beyond HK2 that would preferentially inhibit leukemia while sparing lymphocytes, we compared in vivo proliferating T cells and primary mouse T-ALL cells by RNA-seq. WT bone marrow was transduced with retrovirus expressing a constitutively active Notch protein (Notch1-deltaE) and injected into congenic CD45.1 mice. Mice developed CD4 + CD8+ lymphoblasts in their blood and lymphoid organs. WT primary leukemia cells were isolated from the spleens of recipient mice after 6 weeks and compared to WT CD8+ T cells isolated from mouse spleens 8 days after infection with LCMV (Additional file 3: Figure S3F). A wide range of differentially expressed genes were identified between T cells and leukemic cells (Fig. 9). Three thousand three hundred thirty-one genes were identified as being significantly differentially expressed between T cells and leukemia (Fig. 9a), with leukemic samples enriched in expected gene sets such as Myc targets, beta-catenin pathway, and notch1 signaling (Fig. 9b). T cells were unsurprisingly enriched for gene sets involved in T cell processes such as allograft rejection and IFNγ response (Fig. 9b). Of the differentially expressed genes, 167 were metabolic genes in the KEGG database, including 61 upregulated metabolic genes in leukemia (Fig. 9c). Five of the top metabolic genes upregulated in leukemia when compared to CD8+ T cells were Hmgcs2, Chdh, Fbp1, Prodh, and Ldhb. These genes consistently showed higher expression in leukemia cells when re-evaluated by RT-PCR (Fig. 9d). While some differences were statistically significant after *t* tests, no individual gene reached statistical significance on multiple comparisons correction. Taken together, these data reveal essential differences in T cell and T-ALL cell metabolism that may be harnessed to selectively inhibit leukemic proliferation.

## Discussion

In the past decade, there has been a resurgence of targeting metabolism for cancer therapy. Cancer cells undergo metabolic reprogramming to acquire sufficient nutrients to fuel macromolecule synthesis for growth and proliferation [2, 4, 12]. However, the therapeutic window is limited by metabolic rewiring that cancer cells quickly undergo upon inhibition of a particular metabolic pathway thus becoming resistant to therapies targeting metabolism. Normal proliferating T cells, stem and progenitor cells also display features of metabolic reprogramming similar to cancer cells, further limiting the therapeutic index in targeting metabolism of cancer cells [1, 3, 11]. Moreover, targeting metabolism within T cells to diminish their proliferation and function could leave patients susceptible to life threatening infections while also rendering useless cutting-edge immunotherapies such as checkpoint blockade and CAR-T cells.

A similar metabolic feature of proliferating cancer cells and T cells is that they both highly upregulate glycolytic enzymes [4, 10, 11, 16–18]. HK2 is the one the most highly upregulated glycolytic enzymes in activated T cells and oncogenic transformed cells [16–18, 20, 21]. Thus, we were surprised with our observation that HK2 is dispensable for the essential function of T cells—viral immunity. Clearance of acute LCMV infection was completely intact in mice with *Hk2*^−/−^ T cells, as was the ability of those mice to develop T cell memory. Unexpectedly, we found minimal differences in metabolite levels and gene expression between wild-type and HK2-deficient proliferating CD8 T cells in response to LCMV. Moreover, HK2 deficiency did not impair Treg function. Loss of HK2 also did not impair normal hematopoiesis indicating normal stem and progenitor cell function. Recent work has also shown that HK2 is required for angiogenesis [40], and this inhibition could theoretically further synergize with cancer cell HK2 inhibition to improve response to therapy. In contrast to the observed dispensability of HK2 in immunity, we found that HK2 may play a small role in pathological T cell-mediated inflammation. In a mouse model of colitis, HK2 deficiency conferred a limited degree of protection against inflammation. We speculate that the simplest explanation for this is that pathogenic T cells have a higher demand for glucose which HK2-deficient cells are unable to meet. Indeed, this effect has been observed in other models of inflammation, and 2-DG has been proposed as a potential therapeutic in autoimmune diseases [7, 8]. However, it is also possible the microenvironment specific to these models may play a potential role, and it is also possible that HK2 could have non-metabolic functions contributing to pathogenesis.

It is important to note that recent work from another group supports our finding of HK2 dispensability in T cells, but in a model of herpes virus infection [41]. However, while they propose a compensatory upregulation of HK1- in HK2-deficient T cells, we are unable to confirm that conclusion in our system. There was no observed compensatory increase in HK1 or HK3 expression at the RNA or protein level. Rather, we believe HK2 is dispensable because HK2-mediated glucose phosphorylation only constitutes a small percentage of total glucose phosphorylation in CD4+ and CD8+ T cells.

To identify other targets which could be utilized to selectively inhibit cancer cells, we compared the transcriptome and metabolome of proliferating T cell leukemia with T cells proliferating physiologically in response to infection. This analysis revealed striking differences in how the metabolism of a leukemic cell differs from that of a T cell and identified specific potential targets for cancer therapy, including Hmgcs2, Prodh, Ldhb, Fbp1, and Chdh. This dataset may serve as a significant resource for future studies in cancer therapy to determine if any of the identified highly expressed metabolic genes are also required for leukemia.

## Conclusions

Multiple studies have shown HK2 is essential for cancer growth and development, including in models of breast cancer, lung cancer, and leukemia, leading to interest in HK2 as a potential drug target for cancer therapy. We sought to determine the role of HK2 in T cells, considering the high expression of HK2 in T cells and the potential for HK2 inhibition to adversely affect immunity. We have demonstrated through various animal models that HK2 serves as a mostly redundant enzyme in T cells. We propose that our findings make HK2 an attractive target for cancer therapy, as it would be expected to have limited immunosuppressive side effects. However, future studies are necessary to further investigate the role HK2 inhibition could play in cancer therapy. Testing HK2 dispensability in the context of other viral infections, other immune cell types, and tumor immunity would be important steps to making more generalizable conclusions.

## Additional files

Additional file 1: **Figure S1.** HK isoform expression in Hk2−/− T cells. CD4+ and CD8+ T cells were enriched from splenocytes isolated from adult WT and T-Hk2−/− mice. T cells were activated in vitro with anti-CD3/28 coated beads for 72 h. HK isoform and β-actin protein expression in (A) CD4+ and (B) CD8+ T cells. Biological replicates, *n* = 4. (PDF 5200 kb)

Additional file 2 **Figure S2.** Uncropped Gel Images. HK2 and loading control expression in (A) activated CD4+ T cells and (B) bone marrow. (B) Combined exposure shown or (A) individual exposures shown as appropriate. (PDF 2760 kb)

Additional file 3 **Figure S3.** Example Gating Strategies. (A) Example live CD8+ T cell gating, and subsequent gating for evaluation of activation markers. Similar gating used for CD4+ T cells (B) Gating on single cells for mixed populations of CD4+ and CD8+ T cells followed by gating for CD62L and CD44 expression (performed on CD4+ and CD8+ T cells separately). (C) Gating on live CD4+ cells for expression of transcription factors. (D) Gating on CD8+ cells for gp-33 tetramer. (E) Gating on CD4+ cells for YFP. (F) Gating on single cells for CD45.2 and GFP expression. (PDF 1300 kb)

Additional file 4: **Figure S4.** HK2 is dispensable for T cell function in glucose-limited conditions. Naïve CD4+ and T cells were enriched from splenocytes isolated from adult WT and T-Hk2−/− mice. T cells were activated in vitro with anti-CD3/28 coated beads. (A-B) CD4+ T cells activated in indicated glucose/pyruvate concentrations for 24 h. (B) Percent of CD4+ cells positive for viability and (C) percent of viable CD4+ cells expressing CD69 by flow cytometry. Biological replicates, *n* = 6 from 3 independent experiments (C-E) Enriched splenic CD4+ T cells activated for 72 h in media with 1 mM pyruvate and indicated glucose concentrations. CD4+ T cells analyzed by flow cytometry for (D) percent of cells positive for viability, (E) proliferation as measured by CFSE dilution, and (F) proliferation as measured by cell number. Biological replicates, *n* = 4 WT, 5 T-Hk2−/− from 2 independent experiments. (F-G) Splenocytes were isolated from 8 to 12 week old WT and T-Hk2−/− mice to analyze steady state levels of various populations by indicated marker, *n* = 5 mice. MP = macrophage, DC = dendritic cell, NK = natural killer cell, Neutro = neutrophil. In all panels, error bars represent mean ± SEM, two-way ANOVA with Sidak multiple comparisons correction, *p < 0.05; **p < 0.01. NS = not significant. (PDF 809 kb)

## Abbreviations

2-DG: 2-deoxyglucose
DPI: Days post infection
ECAR: Extracellular acidification rate
Fl: Floxed
HK: Hexokinase
LCMV: Lymphocytic choriomeningitis virus
OCR: Oxygen consumption rate
OVA: Ovalbumin
T-ALL: T cell acute lymphoblastic leukemia
