# Supplementary figures and images for: Hexokinase 2 is dispensable for T cell-dependent immunity
Source: Cancer Metab. 2018 Aug 17;6:10. doi: 10.1186/s40170-018-0184-5 (PMC6098591; doi:10.1186/s40170-018-0184-5)

Figure S1

a

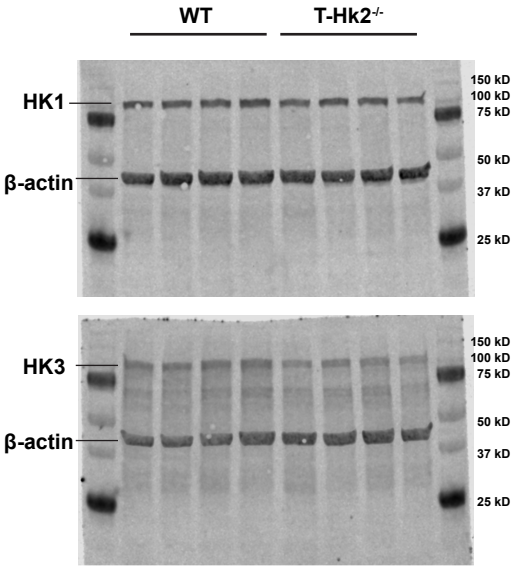

b

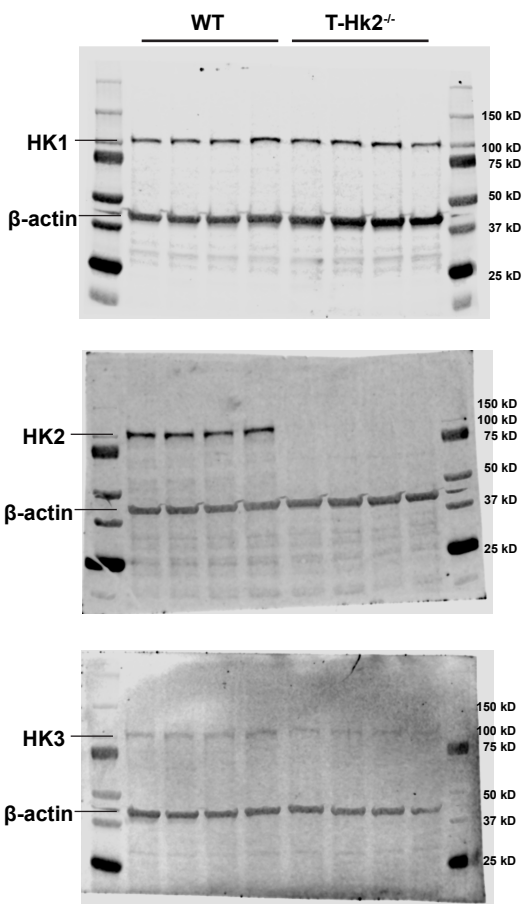

Supplement: Supplementary file 1 — Figure S1. HK isoform expression in Hk2−/− T cells. CD4+ and CD8+ T cells were enriched from splenocytes isolated from adult WT and T-Hk2−/− mice. T cells were activated in vitro with anti-CD3/28 coated beads for 72 h. HK isoform and β-actin protein expression in (A) CD4+ and (B) CD8+ T cells. Biological replicates, n = 4. (PDF 5200 kb) [file 40170_2018_184_MOESM1_ESM.pdf]

Figure S2

a

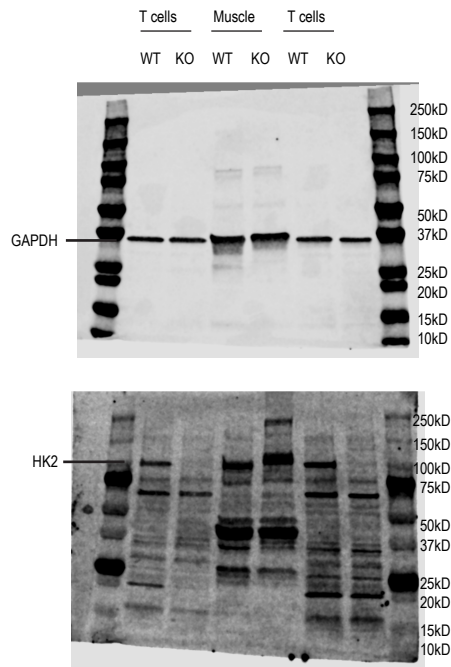

b

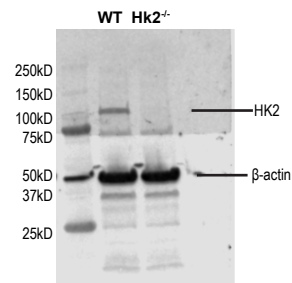

Supplement: Supplementary file 2 — Figure S2. Uncropped Gel Images. HK2 and loading control expression in (A) activated CD4+ T cells and (B) bone marrow. (B) Combined exposure shown or (A) individual exposures shown as appropriate. (PDF 2760 kb) [file 40170_2018_184_MOESM2_ESM.pdf]

**Figure S3**

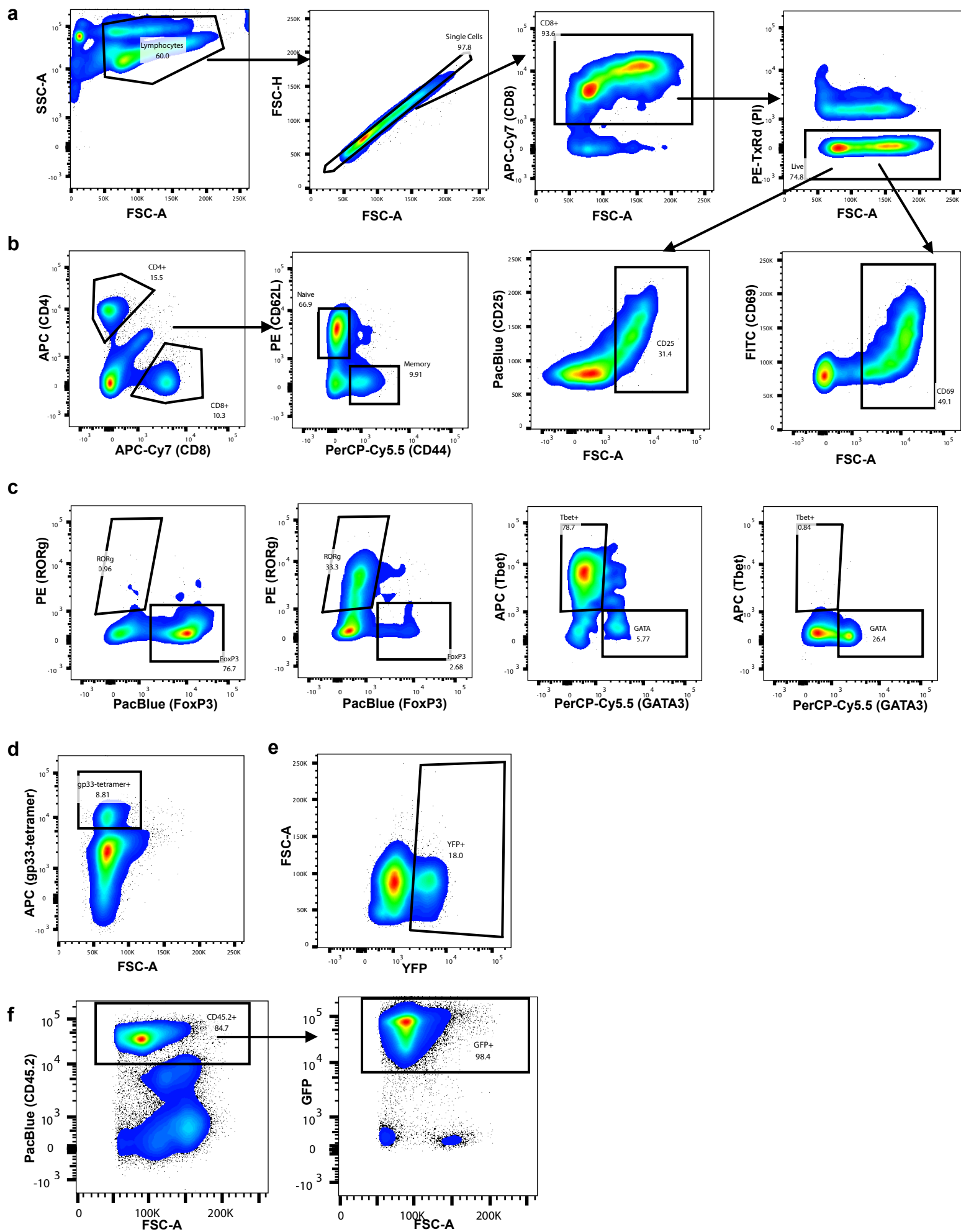

Supplement: Supplementary file 3 — Figure S3. Example Gating Strategies. (A) Example live CD8+ T cell gating, and subsequent gating for evaluation of activation markers. Similar gating used for CD4+ T cells (B) Gating on single cells for mixed populations of CD4+ and CD8+ T cells followed by gating for CD62L and CD44 expression (performed on CD4+ and CD8+ T cells separately). (C) Gating on live CD4+ cells for expression of transcription factors. (D) Gating on CD8+ cells for gp-33 tetramer. (E) Gating on CD4+ cells for YFP. (F) Gating on single cells for CD45.2 and GFP expression. (PDF 1300 kb) [file 40170_2018_184_MOESM3_ESM.pdf]

**Figure S4**

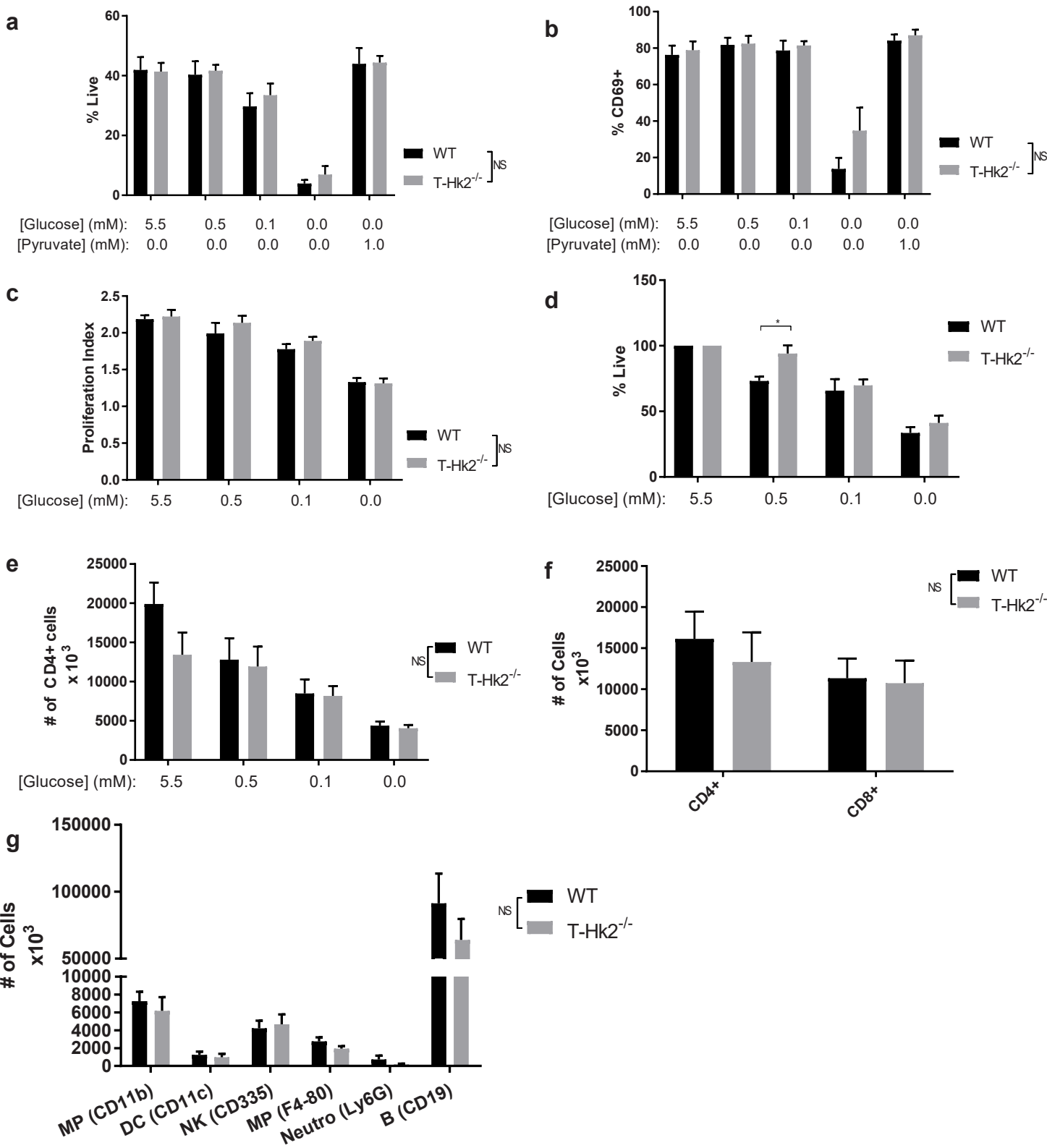

Supplement: Supplementary file 4 — Figure S4. HK2 is dispensable for T cell function in glucose-limited conditions. Naïve CD4+ and T cells were enriched from splenocytes isolated from adult WT and T-Hk2−/− mice. T cells were activated in vitro with anti-CD3/28 coated beads. (A-B) CD4+ T cells activated in indicated glucose/pyruvate concentrations for 24 h. (B) Percent of CD4+ cells positive for viability and (C) percent of viable CD4+ cells expressing CD69 by flow cytometry. Biological replicates, n = 6 from 3 independent experiments (C-E) Enriched splenic CD4+ T cells activated for 72 h in media with 1 mM pyruvate and indicated glucose concentrations. CD4+ T cells analyzed by flow cytometry for (D) percent of cells positive for viability, (E) proliferation as measured by CFSE dilution, and (F) proliferation as measured by cell number. Biological replicates, n = 4 WT, 5 T-Hk2−/− from 2 independent experiments. (F-G) Splenocytes were isolated from 8 to 12 week old WT and T-Hk2−/− mice to analyze steady state levels of various populations by indicated marker, n = 5 mice. MP = macrophage, DC = dendritic cell, NK = natural killer cell, Neutro = neutrophil. In all panels, error bars represent mean ± SEM, two-way ANOVA with Sidak multiple comparisons correction, *p < 0.05; **p < 0.01. NS = not significant. (PDF 809 kb) [file 40170_2018_184_MOESM4_ESM.pdf]
